# The validity of the Physical Literacy in Children Questionnaire in children aged 4 to 12

**DOI:** 10.1186/s12889-024-18343-x

**Published:** 2024-03-21

**Authors:** Yucui Diao, Li Wang, Sitong Chen, Lisa M. Barnett, Emiliano Mazzoli, Inimfon A. Essiet, Xiaofen Wang, Lei Wang, Yaping Zhao, Xuanxi Li, Jing Li

**Affiliations:** 1 School of Physical Education and Sport, Shandong Normal University, No.1, University Road, Changqing district, Jinan, Shandong 250358 China; 2 College of Physical Education, Shandong Agricultural University, Tai’an, China; 3 Institute for Health and Sport, Victoria University, Melbourne, Australia; 4 Institute for Physical Activity and Nutrition (IPAN), School of Health and Social Development, Deakin University, Geelong, Australia; 5 School of Health and Social Development, Deakin University, Geelong, Australia; 6 Centre for Sport, Exercise and Life Sciences, Coventry University, Coventry, UK; 7 Physical Education and Arts School, Chengyi College, Jimei University, Xiamen, China; 8 School of Physical Education, Shanghai University of Sport, Shanghai, China; 9 Library, Shandong Sport University, Jinan, China

**Keywords:** Scale, Self-report, Psychometrics, Child, Measurement equivalence, Cross-cultural validation

## Abstract

**Background:**

Given the growing evidence on the health benefits associated with physical literacy (PL), it is necessary to develop sound measures to assess the levels of PL in children. The Physical Literacy in Children Questionnaire (PL-C Quest) is the first self-report pictorial-based scale to assess children’s perceived PL. It has good validity and reliability in Australian children aged 7 to 12 years, but little is known in younger children and in other cultural contexts. The aim of this study was to examine the validity and reliability in an expanded age range.

**Methods:**

A total of 1,870 Chinese children (girls, *n* = 871; 46.6%), aged 4 to 12 years (*M* = 8.07 ± 2.42) participated in validity testing. Structural equation modeling with the Weighted Least Squares with Mean and Variance approach was used to assess construct validity. The hypothesized theoretical model used the 30 items and four hypothesized factors: physical, psychological, social and cognitive capabilities. Multigroup confirmatory factor analysis was used to assess sex and age group (4–6 years, 7–9 years and 10–12 years) measurement invariance. Internal consistency analyses were conducted using polychoric alpha. A random subsample (*n* = 262) was selected to determine test–retest reliability using Intra-Class Correlations (ICC).

**Results:**

All items except one (moving with equipment–skateboarding) loaded on sub-domains with λ > 0.45. The hypothesized model had a good fit (CFI = 0.954, TLI = 0.950, RMSEA = 0.042), with measurement equivalence across sex and age groups separately. Internal consistency values were good to excellent (overall: α = 0.94; physical: α = 0.86; psychological: α = 0.83; social: α = 0.81; cognitive: α = 0.86). Test–retest reliability was adequate to excellent (overall: ICC = 0.90, physical: ICC = 0.86, psychological: ICC = 0.75, social: ICC = 0.71, cognitive: ICC = 0.72).

**Conclusion:**

The Chinese version of the PL-C Quest is valid and reliable for testing the self-reported PL of Chinese children aged 4 to 12. This study provides the first evidence of validity for this tool in children aged 4–6 years and also evidence that the PL-C Quest would be a meaningful instrument to assess PL in Chinese children.

**Supplementary Information:**

The online version contains supplementary material available at 10.1186/s12889-024-18343-x.

## Introduction

Physical literacy (PL) has emerged as a multidimensional concept in the fields of physical activity, sport, education, and public health internationally [1–3]. PL was defined as “*the motivation, confidence, physical competence, knowledge, and understanding to value and take responsibility for engagement in physical activities for life*” [4]. As a potential factor contributing to health [5], PL is valuable for healthy weight and physical self [6], improved quality of life, well-being and human flourishing [1]. Empirical evidence demonstrates that PL is associated with higher levels of physical activity [7, 8], improved cardiorespiratory fitness [9], and physical and psychosocial well-being [10, 11] in children and adolescents. While children or adolescents with low PL demonstrate low levels of confidence, competence, motivation [6], and physical activity participation [12], they also tend to have higher body weight [13], which further exacerbates the issue of obesity [14]. Given this growing empirical evidence on the benefits and outcomes (including those related to health) associated with PL, it is essential to assess the levels of PL in children.

Despite its health significance, there is still no international agreement on the definition of the concept of PL. Although the definition of the concept of PL by the International Physical Literacy Association (IPLA) [4] has been recognized in some countries [15], other definitions of the concept of PL have emerged and been adapted contextually and culturally. For example, Canadian researchers have operationalized PL into four domains, including affective, physical, cognitive and behavior [16, 17]. In Australia, PL is considered as lifelong holistic learning that integrates skills and attributes spanning four different domains (including physical, psychological, social, cognitive), each containing elements (30 in total) required for lifelong engagement in physical activity [18, 19]. The Australian Physical Literacy Framework (APLF) extends the IPLA’s definition and domains, which has been recognized by many PL researchers [20]. Furthermore, many reviews have found that PL primarily encompasses physical, psychological/affective, cognitive and social domains [15, 21–23]. Thus, the Australian PL definition is arguably one of the most comprehensive to date and also encompasses more psychological and social elements than the IPLA definition; therefore it will be adopted for the present study.

Due to the lack of a globally accepted definition, there is no consensus on the most appropriate approach to assess PL holistically. Current assessments of PL have some weaknesses: 1) the lack of measurement of social domains, which is an important feature of the Australian framework; 2) their inapplicability for young children to self-complete (such as the Canadian Assessment of Physical Literacy, CAPL, Physical Literacy Assessment for Youth, and the Chinese Assessment and Evaluation of Physical Literacy); and 3) the long test time (20–60 min/person), which makes them less suitable for large-scale testing. Although objective measures of PL are often considered a gold standard for some aspects of PL, such as the physical [24], self-assessment (i.e. self-report) is more aligned with the person-centered philosophy of PL [25]. It can provide children, teacher and parents with a comprehensive understanding of the stages of a child’s PL journey [24, 26], and is more suitable to large-scale testing [27]. Thus, a number of self-report instruments have been developed to assess PL in children and adolescents, including the Physical Literacy Assessment for Youth-self (for ages above 7) [28, 29], the Perceived Physical Literacy Instrument for Adolescents (for ages 11–19) [30], the Adolescent Physical Literacy Questionnaire (for ages 12–18) [31], and the Portuguese Physical Literacy Assessment Questionnaire (for ages 15–18) [32]. However, none of the instruments designed for children utilize a pictorial dichotomous format, which would minimize the requirement for written literacy and maximize comprehension of the PL structure.

To address this gap, a pictorial self-reported scale, based on Australia’s PL definition [18, 19] and aligned with the 30 items in the APLF [19], was designed to assess children’s self-perception of their PL [20]. The tool, known as the Physical Literacy in Children Questionnaire (PL-C Quest), is the first pictorial tool for children aged 4 to 12 years old to assess perceptions in four domains (physical, psychological, social and cognitive domain) of PL. In a recent review [33], the PL-C Quest was cited as one of the PL tools with more validity and reliability evidence, and conversely the Chinese tool mentioned earlier was cited as being in the early conceptual stages with no validity and reliability evidence. Although the construct validity, internal consistency and test–retest reliability of the PL-C Quest have been established for Australian children aged 7 to 12 years old [20, 24], no evidence of validity and reliability has been produced for younger children (except for evidence of face validity and response processes) [20, 34] or those from another country with a different cultural context. It is important that an instrument is validated in the intended population and in different cultures in order to facilitate international comparisons. Therefore, the aims of this study were to test the construct validity and reliability (internal consistency and test–retest reliability) of the PL-C Quest in Chinese children aged 4 to 12 years old. Given the mixed evidence for sex measurement invariance of PL-C Quest in Australian children [24], and the lack of evidence for measurement equivalence across age groups, the second purpose was to test the measurement equivalence between sex and across three age groups in Chinese children.

## Methods

### Participants

First, the cities in the northern (Jinan, Shandong Province), central (Shanghai) and southern regions (Xiamen, Fujian Province and Shenzhen, Guangdong Province) in China were selected as these cities reflect different geographical regions in China, which may increase the generalizability of study findings. We then recruited (convenience sample) 10 schools (2 kindergartens and 2 primary schools in Jinan, 1 kindergarten and 1 primary school in each of the three remaining cities). Finally, a total of 10 school principals and 1,870 children aged 4 to 12, along with their parents, agreed to participate in this study. Informed consent was obtained from all subjects involved in the study and their parents or legal guardians. Ethics approval was granted through the Institutional Review Board (IRB) of Shanghai University of Sport (grant number 102772021RT071).

### Translation

Although the best method for cross-cultural adaptation of questionnaires is lacking [35], we translated the PL-C Quest booklets from English to Chinese following the three steps included in most cross-cultural translation guides [36], i.e.: forward translation and synthesis, back-translation, and expert committee review. First, the tool was independently translated from English to Chinese by two experts, one of whom was proficient in English and the other in exercise psychology. Afterwards, the initial Chinese version was developed through discussion between them (step 1). Second, a back translation to English was completed by two other specialists independently, who did not have access to the original English version. Both were native Chinese speakers majoring in English education. Then, the back translated version was approved after they reviewed and discussed any differences in interpretation. After that, the back translated version was sent to the lead author of this tool (Dr Lisa Barnett) to judge whether it accurately reflected the intention of the original English version of the PL-C Quest. Some semantic inaccuracy issues in the back translated version of PL-C Quest were identified by Dr Lisa Barnett (step 2). For instance, item 17 in the back translated version of PL-C Quest literally translated means: “*Some children feel confident to try new sports (e.g., zip line rides)*” and “*Other children do not feel confident trying new sports (e.g., zip line rides)*”. The instrument developer thought that the word “*sports*” did not accurately depict the original version as sports may not include all physical activities. In response, the wording was slightly modified. The language of the final back translated version was: “*Some children feel confident to try new active things (e.g., zip line rides)*” and “*Other children do not feel confident trying new active things (e.g., zip line rides)*”.

**Fig. 1:**
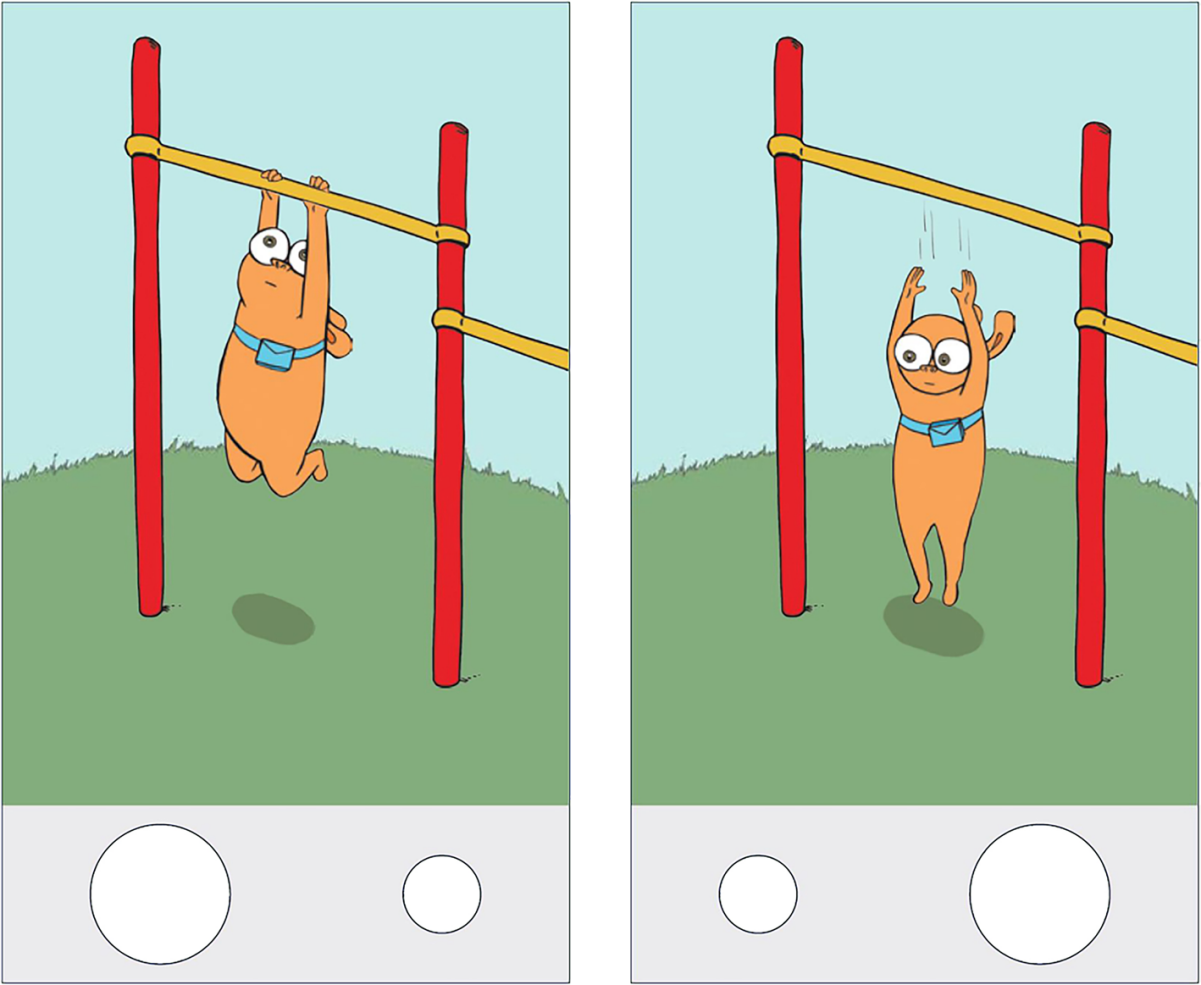
An example of the pictures of the items in the PL-C Quest shown to children. Note. The left picture is the more developed level (i.e. *“some children are pretty good at hanging for a long time without letting go”*); the right one is the less developed level (i.e. *“other children are not so good at hanging for a long time without letting go”*)

Subsequently, the Chinese version was also adjusted in line with the two specialists according to the final back translated version. Third, the final Chinese version was reviewed by a panel of four experts, including two physical education professors and two exercise professors (step 3). A final translated scoring sheet was developed. No changes to the PL-C Quest item drawings (Fig. 1) and scoring system were made.

### Procedure

Children were assessed from October 2022 to January 2023 using the 30 items from the PL-C Quest Chinese version authorized by Sport Australia (now called the Australian Sports Commission). The characters and items in the PL-C Quest were the same for all children. However, the testing procedures are simpler for older children compared to younger children. For young children in kindergarten to Grade 2 (approximately 4- to 8-year-olds), their perceptions of PL were assessed one-on-one in a room with an interviewer. For older children in Grades 3 to 6 (approximately 8- to 12-year-old), one administrator read each scenario out loud and displayed the related images to the students and guided a group of children (20–35 students per group) through a self-completion process. Two weeks later a random subsample of 6 to 8 children from each grade in each school (135 males, 127 females) across four cities were retested to determine test–retest reliability, and the retest procedure was the same as the first test.

The PL-C Quest assesses children’s perceived PL in accordance with the comprehensive APLF, which includes 30 elements within four domains (physical, psychological, cognitive and social). Each item in the PL-C Quest contains two pictures, one representing a more developed level (on the left of the page) and the other representing a less developed level (on the right of the page) (Fig. 1). Children are asked to make two dichotomous choices for each item. For example, to assess item 5 in the physical domain for young children, the evaluator told the children that “*Some children are pretty good at hanging for a long time without letting go*” (by pointing to the picture on the left of the page), “*Other children are not so good at hanging for a long time without letting go*” (by pointing to the picture on the right of the page). Then the children were asked “*Which is more like you?*”. After the child pointed out the picture appropriate for him/her, the child was asked “*Is this picture A LOT like you*” (by pointing to the larger circle below their chosen picture) “*or A BIT like you*” (by pointing to the smaller circle below their chosen picture). The child then proceeded to complete each item in the PL-C Quest using the same dichotomous two-stage process. The testing process for older children was very similar to that described above, but simpler. The older children were required to read the text and look at the central character (a type of rabbit) for each item on their own, and then make a choice via a two-stage dichotomous choice process before putting a cross in the box accordingly. The evaluator walked around and visually checked to ensure that children crossed only one of the four boxes for each item. If children thought both the two pictures were like them, the evaluator asked the child to make a choice based on “*which one of these pictures shows the way you are most of the time?*”.

For scoring, the two options for the ‘more developed’ picture were ‘a lot like me’ (assigned a score of four) or ‘a bit like me’ (three points), while the options for the ‘less developed’ picture were ‘a bit like me’ (two points) or ‘a lot like me’ (one point). Accordingly, perceived PL competence for each item is rated on a 4-point scale. Scores for each item were summed into the overall PL score and subdomain scores (overall: range 30–120; physical: range 12–48; psychological: range 7–28; social: range 4–16; cognitive: range 7–28).

### Data analysis

The sample’s characteristics (sex, age group, city of residences) are presented using descriptive statistics. As the overall PL score and each of the domains were all somewhat negatively skewed, the differences in the overall PL score and each subdomain by child’s sex and age groups (4–6 years, 7–9 years and 10–12 years) were assessed using Mann–Whitney tests and Kruskal–Wallis H tests separately, while the sex differences and age group differences in PL items were assessed using Chi-squared tests. With regard to differences across the three age groups (4–6 years, 7–9 years and 10–12 years), we converted the four options for each item to two dichotomous options to test for differences between groups for enhancing the readability of the results. Descriptive statistics were calculated using IBM SPSS version 26 (IBM Corp., Armonk, NY, USA).

Construct validity was assessed using Confirmatory Factor Analysis (CFA). Due to all items being measured on a 4-point scale, they were analyzed as categorical indicators in the theoretical models. Based on the hypothesized structure of four subdomains and a higher order factor of PL, a Structural Equation Model was conducted (*n* = 1,870) using the Weighted Least Squares with Mean and Variance (WLSMV). Model fit was assessed using: the comparative fit index (CFI), the Tucker–Lewis index (TLI) (values ≥ 0.95 indicative of model fit), and the Root Mean Square Error of Approximation (RMSEA) (values sought between 0 and < 0.06) [37]. Construct validity was assessed using Mplus version 8.3.

Sex and age group measurement invariance were tested by multigroup CFA. For each analysis, configural, metrics, and scalar invariance model were tested. Considering that the χ^2^ statistic is highly sensitive to large samples [38], Δ CFI and ΔRMSEA suggested by Cheung and Rensvold [38] and Meade et al. [39] were used to determine significant sex and age group differences in the models. ΔRMSEA ≤ 0.007 and the ΔCFI ≤ -0.010 indicate measurement invariance between sex and across three age groups (4–6 years, 7-9 years and 10–12 years) [38, 39].

Due to the ordinal nature of the items, internal consistency was assessed using polychoric alphas [40] for all children (*n* = 1,870), and separated by sex and age groups. For ordinal response scales, the polychoric alpha is thought to be a more accurate estimate of reliability than Cronbach’s alpha [41]. Statistical analysis to assess internal consistency and test–retest reliability were conducted in IBM SPSS version 26 (IBM Corp., Armonk, NY, USA) and RStudio Team (version 2022.02.1 Build 461), respectively.

Test–retest reliability (*n* = 262) was calculated using an Intra-Class Correlation (ICC) and separated by sex and age groups [42]. ICC values < 0.5, 0.5–0.75, 0.75–0.9, and > 0.9 indicated poor, moderate, good, and excellent reliability, respectively [43].

## Results

### Demographic characteristics and descriptive statistics

Table 1 shows the demographic characteristics of the subjects. A total of 1,870 children (female, *n* = 871; 46.58%), 4 to 12 years of age (*M* = 8.07, *SD* = 2.42) participated in this study. The proportions of children aged 4–6, 7–9, and 10–12 are 36.15%, 34.97%, and 28.88%, respectively. The children reside in different provinces, with 42.99% in Shandong Province, 25.94% in Fujian Province, and the remaining distributed between Guangdong Province and Shanghai. The test–retest sample were 262 children (female, *n* = 127; 48.47%) with a mean age of 8.17 (*SD* = 2.42). The overall PL score and each of the domains were all somewhat negatively skewed. Table 2 presents summarized statistics of the overall and subdomain scores, and by sex and age groups. Supplementary Tables 1 and 2 show the percentage of children at each of the four PL levels (1–4) for each item, and by sex and age group separately.

**Table 1:** Demographic characteristics of the samples

Characteristic	Total Sample	Test–retest Sample
Children,* n*	1870	262
Age, M (SD)	8.07 (2.42)	8.17 (2.42)
Age group, *n* (%)		
4–6 years	676 (36.15)	95 (36.26)
7–9 years	654 (34.97)	91 (34.73)
10–12 years	540 (28.88)	76 (29.01)
Sex *n* (%)		
Male	999 (53.42)	135 (51.53)
Female	871 (46.58)	127 (48.47)
Province of residence (China), *n* (%)		
Shandong (Jinan)	804 (42.99)	125 (47.71)
Fujian (Xiamen)	485 (25.94)	57 (21.75)
Guangdong (Shenzhen)	339 (18.13)	40 (15.27)
Shanghai	242 (12.94)	40 (15.27)

**Table 2:** Overall physical literacy and subdomain scores for all children, and by sex (girls, *n* = 871; boys, *n* = 999) and age groups (4–6 years, *n* = 676; 7–9 years, *n* = 654; 10–12 years, *n* = 540)

Domain	Total		Girls		Boys		Girls VS Boys		A		B		C		Age group differences		Post-hoc
	M (SD)	Mdn	M (SD)	Mdn	M (SD)	Mdn	Z	*P*	M (SD)	Mdn	M (SD)	Mdn	M (SD)	Mdn	H	*P*	
Physical (range 12–48)	37.2 (7.0)	38.0	36.5 (6.7)	37.0	37.8 (7.1)	39.0	-4.45	0.000	36.7 (7.2)	37.0	38.6 (6.4)	39.0	36.0 (7.1)	36.5	46.37	0.000	B > C; B > A
Psychological (range 7–28)	23.6 (3.9)	24.0	23.3 (3.8)	24.0	23.8 (3.9)	25.0	-3.57	0.000	22.9 (4.0)	23.0	24.2 (3.6)	25.0	23.5 (3.9)	24.0	36.77	0.000	B > C; B > A
Social (range 4–16)	13.8 (2.4)	14.0	13.8 (2.3)	14.0	13.9 (2.4)	15.0	-1.19	0.236	13.9 (2.4)	15.0	14.1 (2.2)	15.0	13.4 (2.5)	14.0	28.76	0.000	A > C; B > C
Cognitive (range 7–28)	24.2 (3.8)	25.0	24.1 (3.6)	25.0	24.4 (3.9)	25.0	-3.01	0.003	23.4 (4.1)	24.0	24.9 (3.5)	26.0	24.5 (3.4)	25.0	51.16	0.000	B > C; C > A; B > A
Overall (range 30–120)	98.8 (14.3)	100.0	97.7 (13.7)	99.0	99.8 (14.7)	102.0	-4.01	0.000	97.0 (14.7)	97.5	101.9 (13.4)	104.0	97.4 (14.2)	99.5	48.72	0.000	B > C; B > A

A = 4 to 6 years. B = 7 to 9 years. C = 10 to 12 years. Mann–Whitney test and Kruskal–Wallis H tests was used to calculate sex-based differences and age group differences for the overall and subdomain scores separately

*M* Mean, *SD* standard deviation, *Mdn* Median

In relation to sex differences (Table 2), Mann–Whitney tests revealed that boys scored higher than girls in terms of the overall PL score and three of the four subdomains (*N*_boy_ = 999, *N*_girl_ = 871; Overall: z = –4.01, *p* < 0.001; Physical: z = –4.45, *p* < 0.001; Psychological: z = –3.57, *p* < 0.001; Cognitive: z = –3.01, *p* = 0.003); but not the social domain (z = –1.19, *p* = 0.236). Supplementary Table 1 shows the sex difference results for each of the PL items.

In terms of age-group differences (Table 2), Kruskal–Wallis H tests showed significant age-group differences in both the overall PL score and subdomains (*N*_4-6_ = 676, *N*_7-9_ = 654, *N*_10-12_ = 540; overall: H = 48.72, *p* < 0.001; physical: H = 46.37, *p* < 0.001; psychological: H = 36.77, *p* < 0.001; social: H = 28.76, *p* < 0.001; cognitive: H = 51.16, *p* < 0.001). Post hoc comparisons revealed that children aged 7 to 9 scored higher than children aged 4–6 and 10–12 years in the overall PL score and three subdomains score (physical domain, psychological domain and cognitive domain), and children aged 10–12 years scored higher than children aged 4–6 years in the cognitive domain. Additionally, children aged 10–12 years scored lower than children aged 4–6 and 7–9 years in the social domain. Supplementary Figs. 1 to 4, and Supplementary Table 2 shows the chart of responses (level 1–4, %) and the post-hoc comparison results for each PL item in the four domains by age groups respectively.

### Construct validity

**Fig. 2:**
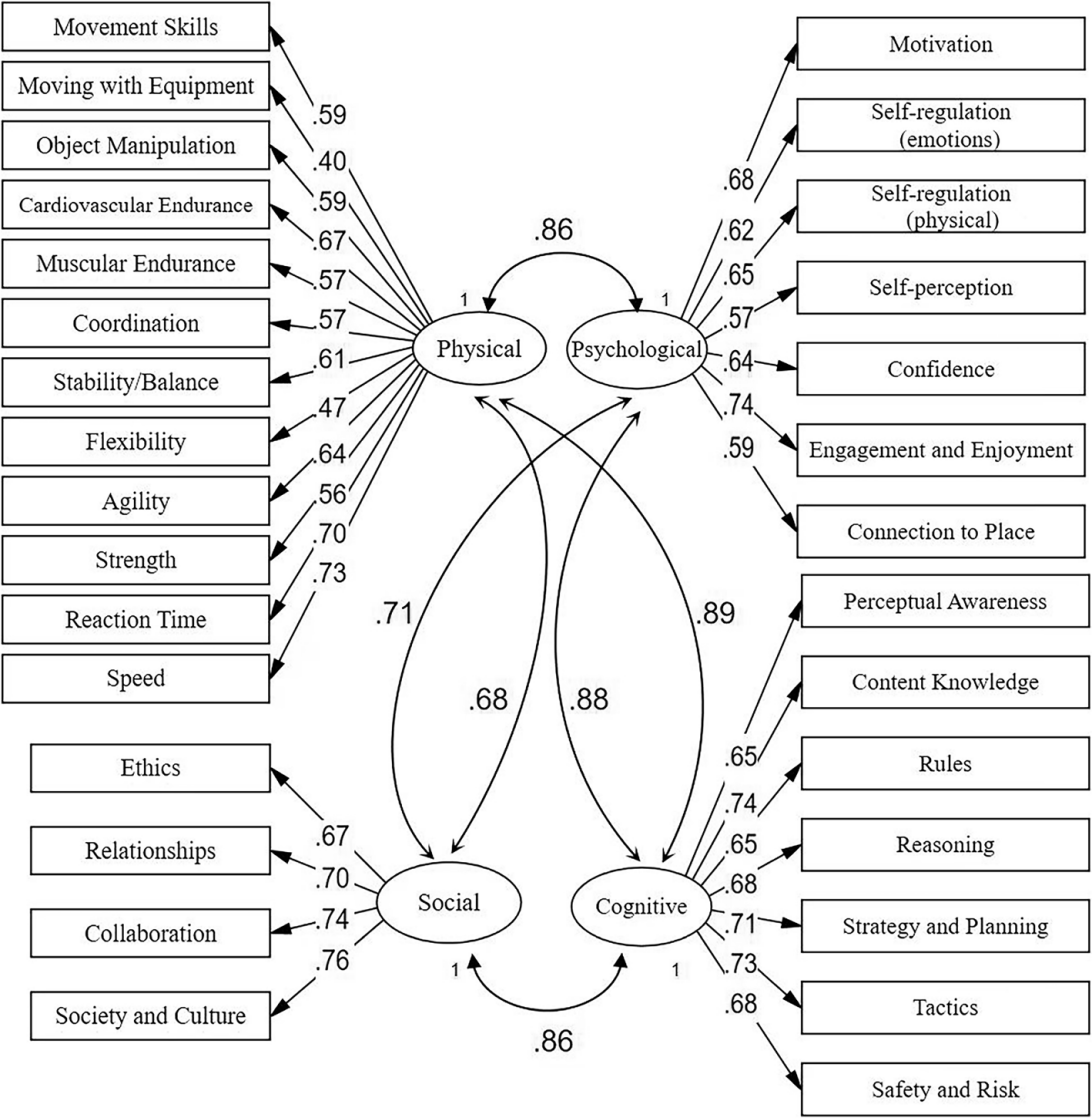
Hypothesized model of PL-C Quest with item loadings with robust weighted least square mean and variance adjusted, WLSMV

Figure 2 shows the theoretical model with item loadings. Items typically loaded on sub-domains close or above the standard of λ = 0.45 [44], except for *moving with equipment*–skateboarding (λ = 0.40). Supplementary Table 3 shows a good fit of the theoretical model to the data for the total sample (CFI = 0.954, TLI = 0.950, RMSEA = 0.042), between sex (boys: CFI = 0.968, TLI = 0.965, RMSEA = 0.036; girls: CFI = 0.941, TLI = 0.936, RMSEA = 0.045) and across age groups (4–6 years: CFI = 0.929, TLI = 0.923, RMSEA = 0.046; 7–9 years: CFI = 0.968, TLI = 0.965, RMSEA = 0.035; 10–12 years: CFI = 0.956, TLI = 0.952, RMSEA = 0.047).

Multigroup analysis were conducted for measurement invariance between sex and across three age groups. For sex measurement invariance, all the ΔCFI (0 for metrics invariance, -0.008 for scalar invariance) and ΔRMSEA (0 for metrics invariance, 0.002 for scalar invariance) in the models did not exceed the cut off values (ΔRMSEA ≤ 0.007 or the ΔCFI ≤ -0.010), indicating the PL-C Quest had measurement equivalence between the sexes. For age group measurement invariance, the ΔCFI (ΔCFI = -0.002) and ΔRMSEA (ΔRMSEA = 0) for metrics invariance in the models did not exceed the cut off values (ΔRMSEA ≤ 0.007 or the ΔCFI ≤ 0.010), indicating the true factor loadings are equal for the three age groups. The ΔRMSEA for scalar invariance (ΔRMSEA = 0.004) was below the cut off values, whereas the ΔCFI (ΔCFI = -0.015) slightly exceeded the cut off value i.e., 0.010. This suggests that the strong measurement equivalence of PL-C Quest across age groups is not supported and indicates that item intercepts differ across age groups of children, i.e., that differences exist across age groups. However, the figural and item factor loadings of PL-C Quest are equivalent across age groups, suggesting that the PL-C items have the same significance and function across age groups. Complete multigroup analysis across sex and age groups results are reported in Table 3.

**Table 3:** Multiple-group analysis across sex and age group of the PL-C quest

Model	χ^2^	*df*	CFI	TLI	RMSEA [90%CI]	△CFI	△RMSEA
***Sex***							
M_0_ (configural)	2016.035	798	0.957	0.953	0.040 [0.038, 0.043]		
M_1_ (metric)	2039.665	824	0.957	0.955	0.040 [0.038, 0.042]	0.000	0.000
M_2_ (scalar)	2314.977	880	0.949	0.950	0.042 [0.040, 0.044]	-0.008	0.002
***Age group***							
M_0_ (configural)	2576.32	1197	0.952	0.948	0.043 [0.041, 0.045]		
M_1_ (metric)	2683.57	1249	0.950	0.948	0.043 [0.041, 0.045]	-0.002	0.000
M_2_ (scalar)	3253.82	1361	0.935	0.937	0.047 [0.045, 0.049]	-0.015	0.004

Probability level *p* < 0.05. Configural = whether factor structure is same across groups; Metric = whether factor loadings are the same across groups; Scalar = whether intercepts or thresholds are the same across groups

*χ*^*2*^ chi-square, *df* degrees of freedom, *CFI* comparative fit index, *TLI*, Tucker-Lewis index, *RMSEA* Root Mean Square Error of Approximation, *CI* confidence interval

### Reliability

Internal consistency values were good to excellent (overall: α = 0.94; physical: α = 0.86; psychological: α = 0.83; social: α = 0.81; cognitive: α = 0.86). Boys had slightly higher polychoric alpha values than girls for the total scale (0.95 vs 0.93), physical (0.88 vs 0.84), psychological (0.85 vs 0.80), social (0.82 vs 0.79) and cognitive domains (0.88 vs 0.84). Separate analysis by age group showed that polychoric alpha values for children aged 10 to 12 years (overall: α = 0.94; physical: α = 0.87; psychological: α = 0.85; social: α = 0.83; cognitive: α = 0.87), aged 7 to 9 years (overall: α = 0.95; physical: α = 0.86; psychological: α = 0.84; social: α = 0.81; cognitive: α = 0.88), and aged 4 to 6 years were very similar (overall: α = 0.93; physical: α = 0.86; psychological: α = 0.80; social: α = 0.78; cognitive: α = 0.84). Complete internal consistency reliability results are reported in Table 4.

**Table 4:** Internal consistency reliability with polychoric alpha (with 95% confidence interval) and divided by sex and age group

Domain	Total *n* = 1870	Sex		Age group		
		Boy *n* = 999	Girl *n* = 871	A *n* = 676	B *n* = 654	C *n* = 540
Sum of domains (30 items) Range 30 to 120	0.94 [0.91, 0.97]	0.95 [0.92, 0.97]	0.93 [0.89, 0.96]	0.93 [0.89, 0.96]	0.95 [0.92, 0.97]	0.94 [0.91, 0.97]
Physical (12 items) Range 12 to 48	0.86 [0.71, 0.95]	0.88[0.75, 0.96]	0.84 [0.66, 0.94]	0.86 [0.70, 0.95]	0.86 [0.70, 0.95]	0.87 [0.73, 0.96]
Psychological (7 items) Range 7 to 28	0.83 [0.53, 0.97]	0.85[0.58, 0.97]	0.80 [0.45, 0.96]	0.80 [0.46, 0.96]	0.84 [0.55, 0.97]	0.85 [0.59, 0.97]
Social (4 items) Range 4 to 16	0.81[0.01, 0.99]	0.82 [0.07, 0.99]	0.79 [-0.05, 0.99]	0.78 [-0.11, 0.98]	0.81 [0.01, 0.99]	0.83 [0.13, 0.99]
Cognitive (7 items) Range 7 to 28	0.86[0.62, 0.97]	0.88 [0.67, 0.98]	0.84 [0.56, 0.97]	0.84 [0.56, 0.97]	0.88 [0.67, 0.98]	0.87 [0.63, 0.97]

A = 4 to 6 years. B = 7 to 9 years. C = 10 to 12 years

A total of 262 children (51.5% boys) aged from 4.25 to 12.58 years (*Mean* = 8.17 years, *SD* = 2.42) completed the survey twice two weeks apart. Test–retest values were excellent for the total scale (ICC = 0.90, 95% confidence interval (CI) [0.86, 0.94]), good for the physical domain (ICC = 0.86, 95% CI [0.81, 0.89]), and moderate to good for the psychological (ICC = 0.75, 95% CI [0.66, 0.81]), social (ICC = 0.71, 95% CI [0.63, 0.78]) and cognitive domains (ICC = 0.72, 95% CI [0.64, 0.78]). Boys had higher ICC values than girls for the total scale (0.91 vs 0.85), the physical (0.88 vs 0.79) and psychological (0.78 vs 0.70), but similar ICC values to girls in the social (0.71 vs 0.72) and cognitive domains (0.70 vs 0.74). The ICC values of the total scale and four subdomains in the three age groups are all higher than 0.60, with the highest value for children aged 10 to 12 years (overall: ICC = 0.94; physical: ICC = 0.89; psychological: ICC = 0.83; social: ICC = 0.82; cognitive: ICC = 0.78), followed by children aged 7 to 9 years (overall: ICC = 0.93; physical: ICC = 0.88; psychological: ICC = 0.80; social: ICC = 0.75; cognitive: ICC = 0.72), and the lowest value for children aged 4 to 6 years (overall: ICC = 0.83; physical: ICC = 0.81; psychological: ICC = 0.64; social: ICC = 0.60; cognitive: ICC = 0.64). Complete test–retest reliability results are reported in Table 5.

**Table 5:** Test–retest reliability results for children (*n* = 262) and divided by sex and age group

Physical Literacy Domains	Test 1				Test 2				Test–retest Reliability		
**All *n* = 262**	Min	Max	M	SD	Min	Max	M	SD	ICC	95%LCI	95%UCI
Sum of domains	34	120	100.77	14.50	37	120	102.24	13.41	0.90	0.86	0.94
Physical	13	48	37.94	7.47	17	48	38.71	6.64	0.86	0.81	0.89
Psychological	7	28	24.10	3.70	8	28	24.35	3.69	0.75	0.66	0.81
Social	4	16	14.05	2.23	4	16	14.14	2.28	0.71	0.63	0.78
Cognitive	7	28	24.69	3.49	8	28	25.04	3.24	0.72	0.64	0.78
***Sex***											
**Boys *n* = 135**											
Sum of domains	58	120	103.00	14.26	62	120	103.59	13.50	0.91	0.87	0.94
Physical	13	48	39.13	7.63	17	48	39.50	6.93	0.88	0.82	0.92
Psychological	13	28	24.55	3.59	14	28	24.74	3.40	0.78	0.68	0.86
Social	7	16	14.25	2.02	5	16	14.20	2.24	0.71	0.59	0.81
Cognitive	16	28	25.07	3.16	15	28	25.16	3.03	0.70	0.59	0.80
**Girls *n* = 127**											
Sum of domains	34	120	98.41	14.44	37	120	100.80	13.21	0.85	0.78	0.91
Physical	15	48	36.66	7.11	17	48	37.88	6.24	0.79	0.69	0.86
Psychological	7	28	23.63	3.77	8	28	23.93	3.95	0.70	0.56	0.81
Social	4	16	13.83	2.42	4	16	14.07	2.32	0.72	0.59	0.81
Cognitive	7	28	24.28	3.78	8	28	24.91	3.45	0.74	0.64	0.82
***Age group***											
**4–6 years *n* = 95**											
Sum of domains	58	120	99.13	13.68	64	120	102.64	12.40	0.83	0.73	0.90
Physical	20	48	37.23	7.54	24	48	38.91	6.43	0.81	0.72	0.88
Psychological	15	28	23.75	3.46	13	28	24.44	3.48	0.64	0.48	0.79
Social	7	16	14.11	2.16	6	16	14.46	2.07	0.60	0.45	0.73
Cognitive	12	28	24.04	3.30	11	28	24.83	3.32	0.64	0.49	0.77
**7–9 years *n* = 91**											
Sum of domains	34	120	103.34	13.92	37	120	104.54	12.41	0.93	0.88	0.95
Physical	16	48	39.35	6.46	17	48	40.11	5.60	0.88	0.80	0.94
Psychological	7	28	24.46	3.70	8	28	24.57	3.52	0.80	0.68	0.88
Social	4	16	14.24	2.00	4	16	14.26	2.06	0.75	0.63	0.84
Cognitive	7	28	25.29	3.58	8	28	25.59	3.12	0.72	0.56	0.84
**10–12 years *n* = 76**											
Sum of domains	58	120	99.76	15.90	62	120	98.97	15.18	0.94	0.88	0.96
Physical	13	48	37.12	8.31	17	48	36.80	7.60	0.89	0.80	0.94
Psychological	13	28	24.12	3.98	13	28	23.96	4.13	0.83	0.70	0.91
Social	6	16	13.75	2.54	5	16	13.58	2.66	0.82	0.70	0.92
Cognitive	13	28	24.78	3.52	14	28	24.63	3.23	0.78	0.65	0.87

*M* Mean, *SD* standard deviation, *ICC* intraclass correlation coefficient, *LCI* lower confidence interval, *UCI* upper confidence interval

## Discussion

The PL-C Quest is the only existing self-report pictorial-based scale to assess perceived PL in children, with face and content validity for children from 5–12 years old [20, 34] and other aspects of reliability and validity in Australian children aged 7 to 12 years old [20, 24]. To verify the applicability of PL-C Quest in a younger age group and in China, we tested the psychometric characteristics of the PL-C Quest in Chinese children aged 4–12 years. We found that the construct validity and reliability (internal consistency and test–retest) of the PL-C Quest were good in Chinese children. To our knowledge, this is the first study to test the psychometric properties of PL-C Quest in a culture other than the one in which it was developed, which not only provides preliminary evidence of its applicability in Chinese culture but also broadens its age applicability. Further, it also provides a new approach to measure PL reliably and validly using a self-report pictorial-based perspective at a large-scale level.

### Validity

The hypothesized model of the PL-C Quest, with four factors (i.e., physical, psychological, social and cognitive), was supported with an excellent fit in Chinese children aged 4 to 12 years. Similar results were documented in Australian children aged 7–12 years [24]. Unlike the Australian study [24], we tested the construct validity of PL-C Quest with WLSMV rather than the maximum likelihood approach. In general, WLSMV estimated factor loadings and robust standard errors more accurately than maximum likelihood especially as there were less than 5 categories [45]. This may explain why our fit indicators were better than those found in Australian children [24].

At the item level, consistent with the results of the Australian study [24], we also found that the item, *moving with equipment* (skateboarding), did not load to the physical domain at the minimum required level (λ = 0.40) [44]. This may be due to the fact that children in both China and Australia rated themselves lower on this item compared to other items of the physical domain. Previous research has identified that children have lower self-perceived motor skills in skills they have not attempted compared to proficient skills [46–48]. In fact, skateboarding has only recently been included in the ‘Curriculum Standards for Physical Education and Health in Compulsory Education (2022 Edition)’ [49] – a national standard that guides physical education for primary and secondary school students in China. As such, skateboarding is not yet as common in China as other motor skills in the physical domain, which may result in a lack of practice experience and thus a lower self-perception of this item for most Chinese children. In light of this and the fact that it will be an official Olympic event, future research could further investigate whether Chinese children improve in their self-perception of skateboarding.

A vital contribution of this study is our examination of whether the PL-C Quest has the same meaning and potential structure in different population groups (sex and age). In terms of sex, results indicated that the factor loadings and thresholds of the PL-C Quest were equivalent between boys and girls in Chinese children, thus suggesting that the model did not vary by sex. A similar finding was reported in Australian children based on the value of alternative fit indices (ΔCFI andΔRMSEA) [24]. This highlights that the PL-C Quest has measurement equivalence across sex. Notably, inclusivity in terms of gender identities was given precedence by the expert group in the development of the PL-C Quest. Instead of depicting genders in the pictorial scale, a ‘rabbit—bunny’ character that was gender neutral, was developed to demonstrate each item [20]. This approach may have contributed to sex equivalent findings for the PL-C Quest.

Regarding the age group, this study supports the notion that the PL-C Quest possesses the same potential structure across early, middle, and late childhood in China. However, strong measurement invariance of age groups in the PL-C Quest was not fully confirmed which may be due to expected age-related factors. Children’s physical activity experience and cognitive abilities improve as they grow up, which may lead to a more accurate self-perception of items in the PL-C Quest. To accommodate the limited cognitive ability and motor experience of younger children, it is recommended that any items not easily understood by children could be appropriately explained during the test based on the essential meaning of each item in PL-C Quest.

Overall, the scale can be used to measure the characteristics of PL in children aged 4 -12 years and to examine the development of children’s PL in China. The cultural diversity was also given precedence by the expert group in the development of PL-C Quest. The ‘rabbit—bunny’ character that demonstrates each item of PL-C Quest was not representative of a particular race or ethnicity and appealing to children [20], which may contribute to the better validity of PL-C Quest among Chinese children.

### Reliability

Similar to Australian children [24], the internal consistency values were also good for Chinese children. In this study, Chinese boys reported higher levels of PL, which may lead to slightly better internal consistency reliability among boys compared to girls. The internal consistency reliability was also good for the three age groups.

Overall, the test–retest reliability was adequate to good for the overall PL score and four subdomain scores in Chinese children, similar to Australian children [24]. Additionally, we further tested the test–retest reliability of the PL-C Quest by sex and age groups. Similar to Australian children [24], the ICC values for Chinese boys were also higher than Chinese girls in the overall PL score and the physical and psychological domain scores, suggesting that boys’ self-perceptions are more stable and reliable. This may be due to the fact that boys generally have higher physical competence [50] and are more consistent and persistent in their enjoyment of physical activity than girls [51]. However, unlike the results of the Australian study [24], we found little difference between boys’ and girls’ ICC values in the social and cognitive domains in China, which may be explained by cultural differences. In China, children’s social skills and academic achievement are areas of particular importance to parents and teachers [52]. Notably, the collectivist culture emphasizes children’s cooperation with others and integration into the group in the socialization practices [53], which may contribute to the fact that the identification of such cultural values in physical activity may not change significantly over time for both Chinese boys and girls. Furthermore, three to four physical education and health lessons per week [54] ensures sufficient time to learn and review knowledge of physical activity for children in primary school, and this may lead to more stable and reliable cognition of PL for both boys and girls.

It was also noted that test–retest reliability increased with the age of children, which may be accounted for the child’s growing cognitive abilities. Children’s self-perceptions become progressively more accurate from about age eight [55, 56], which may have led to a higher test–retest reliability in older children. In summary, the PL-C Quest is a reliable scale to test the PL of Chinese children aged 4 to 12 years.

### Self-reported physical literary between boys and girls

For the overall PL score, we found Chinese boys had higher scores than girls due to the greater physical, psychological and cognitive self-perceived level they presented, which reflects the literature in terms of actual PL scores assessed by CAPL-2 in Hong Kong children aged 8 to 12 years [57]. This may suggest that both the actual and self-perceived PL is better in Chinese boys than in girls. In terms of sex differences in each domain, Chinese boys have higher levels of perceived motor competence in preschool [58] and primary school [59], which confirm our findings. Children with higher perceived physical competence are more likely to experience higher levels of physical activity enjoyment [51] and motivation [60, 61], and in our sample, Chinese boys had higher perceived physical and psychological competence than girls. The PL-C Quest contained elements of *strategy*, *reasoning*, *tactics* in physical activity and sport that boys may have more experience with, which may have led to the higher perceived cognitive competence of PL by Chinese boys. No sex difference was found for the social domain in our sample of Chinese children. In fact, the non-sex differences in the social competence of Chinese children aged 10–12 years [62] supports our finding.

### Self-reported physical literary across the age groups

Significant age group differences were found for the overall, domain and item scores of PL in Chinese children, with children in middle childhood (aged 7–9 years) scoring highest on the overall PL scores and two subdomain scores (physical and psychological domains). This may suggest that the development of self-perceived PL, physical competence and psychological competence in childhood follows a slightly inverted U-shape, i.e., a gradual increase from early to middle childhood and decrease from late childhood. While it is generally accepted that younger children below age 8 have a more positive bias on self-perceptions [56], our results show that the positive bias for physical and psychological domain of PL is not highest in the children aged 4–6 years, but rather in the children aged 7–9 years, which can be explained by children’s physical development and growth of movement experiences.

Since the items on the PL-C Quest are mainly focused on movement and physical activity, and that children who have tried certain movements have a higher perception of physical competence [47, 63], it is possible that at this level, children aged 4–6 years may have a lower perception of physical competence and psychological competence due to a lower level of physical development and less movement experience compared to children aged 7–9 years. This finding is consistent with a previous study that also found better perceived physical competence in Chinese children aged 7–9 year compared to those aged 4–6 years [59]. It is not until late in childhood that children’s ability to use social comparison and to differentiate real from ideal self-perceptions gradually increases, leading to a more accurate self-perception. Meanwhile, the dichotomous pictures of comparison set up by the PL-C Quest may have made it easier for children to select more accurate answers through comparison. Therefore, children’s perception of the overall PL, physical and psychological domain scores appear to decrease in later childhood following the developmental trend of the self-concept in childhood and adolescents [64, 65].

Social competence, one of the dimensions of self-perception, was lowest in children aged 10–12 years compared to that in early and middle childhood, which is in line with children’s social self-perception development. On the other hand, cognitive domain scores increased with the age group, suggesting that children’s perceived knowledge and understanding of movement and physical activity gradually improved with cognitive development. It is worth noting that an Australian study found non-significant correlations between perceived PL and age for children aged 7 to 12 years [24], but this might be explained by the smaller age range. Further work is needed to identify age differences in children’s perceived PL throughout childhood, including those in early childhood, as well as cross-cultural differences in such differences.

### Strengths and limitations

The first strength of this study lies in the inclusion of children in the younger age group (4–6 years) and the confirmation of good construct validity and reliability of the PL-C Quest in early, middle, and late childhood, which broadens the age range of children to which the instrument can be applied. Another strength is the large sample size, the methods and statistical approach (WLSMV) to determine construct validity are advanced and appropriate.

The main limitation of this study is the lack of the criterion validity for PL-C Quest as there is no ‘gold standard’ of PL available yet [24]. Future studies could select widely used self-report questionnaires in the physical, psychological, social and cognitive domains of PL for further validation. Another limitation is that we were unable to use random sampling in selecting cities due to limited funding, compromising the representativeness of the sample. Therefore, future research is needed to assess the reliability and validity using a representative sample. Future studies should concentrate on the longitudinal development of perceived PL in children and adolescents, as well as cross-cultural comparisons of perceived PL in this population. Our study confirmed the PL-C Quest as a new and suitable measure for large-scale testing. This will facilitate the development of personalized intervention programs aimed at enhancing their perceived PL.

## Conclusion

This study provides valuable information on the validity and reliability of PL-C Quest not only in Chinese culture, but also in young children aged 4 to 6 years of age. The outcomes verify that the Chinese version of the PL-C Quest is a reliable and valid pictorial scale for testing the PL of Chinese children aged 4 to 12.

### Supplementary Information

**Supplementary Material 1.**

## Data Availability

The data that support the findings of this study are not openly available due to reasons of sensitivity and are available from the corresponding author upon reasonable request.

## Abbreviations

APLF: Australian Physical Literacy Framework
CAPL: Canadian Assessment of Physical Literacy
CFI: Comparative Fit Index
CI: Confidence Interval
CFA: Confirmatory Factor Analysis
ICC: Intra-Class Correlation
IPLA: International Physical Literacy Association
PL: Physical literacy
PL-C Quest: Physical Literacy in Children Questionnaire
RMSEA: Root Mean Square Error of Approximation
TLI: Tucker–Lewis Index
WLSMV: Weighted Least Squares with Mean and Variance
