# Supplementary material for: The validity of the Physical Literacy in Children Questionnaire in children aged 4 to 12
Source: BMC Public Health. 2024 Mar 21;24:869. doi: 10.1186/s12889-024-18343-x (PMC10956319; doi:10.1186/s12889-024-18343-x)
Supplement: Supplementary file 1 — Supplementary Material 1. [file 12889_2024_18343_MOESM1_ESM.zip › Supp_fig 1 to 4.docx]

**Supplementary Figure 1**

*Chart of responses (level 1-4, %) for each physical literacy items in the physical domain by age groups*

**
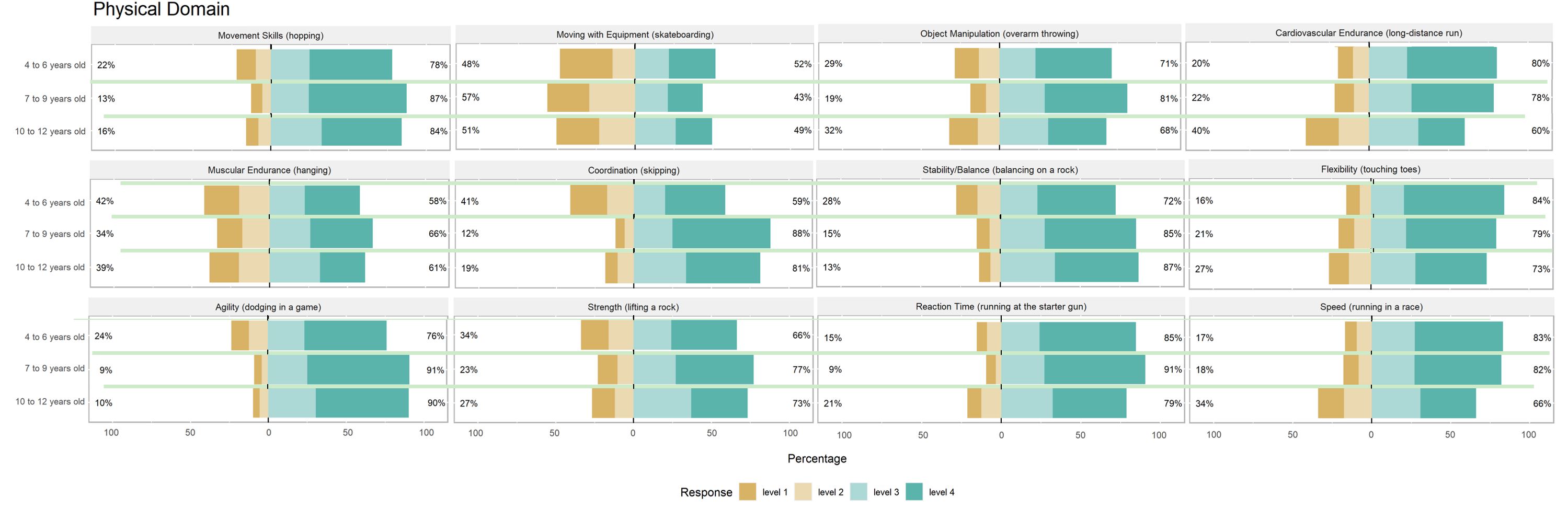
**

**Supplementary Figure 2**

*Chart of responses (level 1-4, %) for each physical literacy items in the psychological domain by age groups*

**
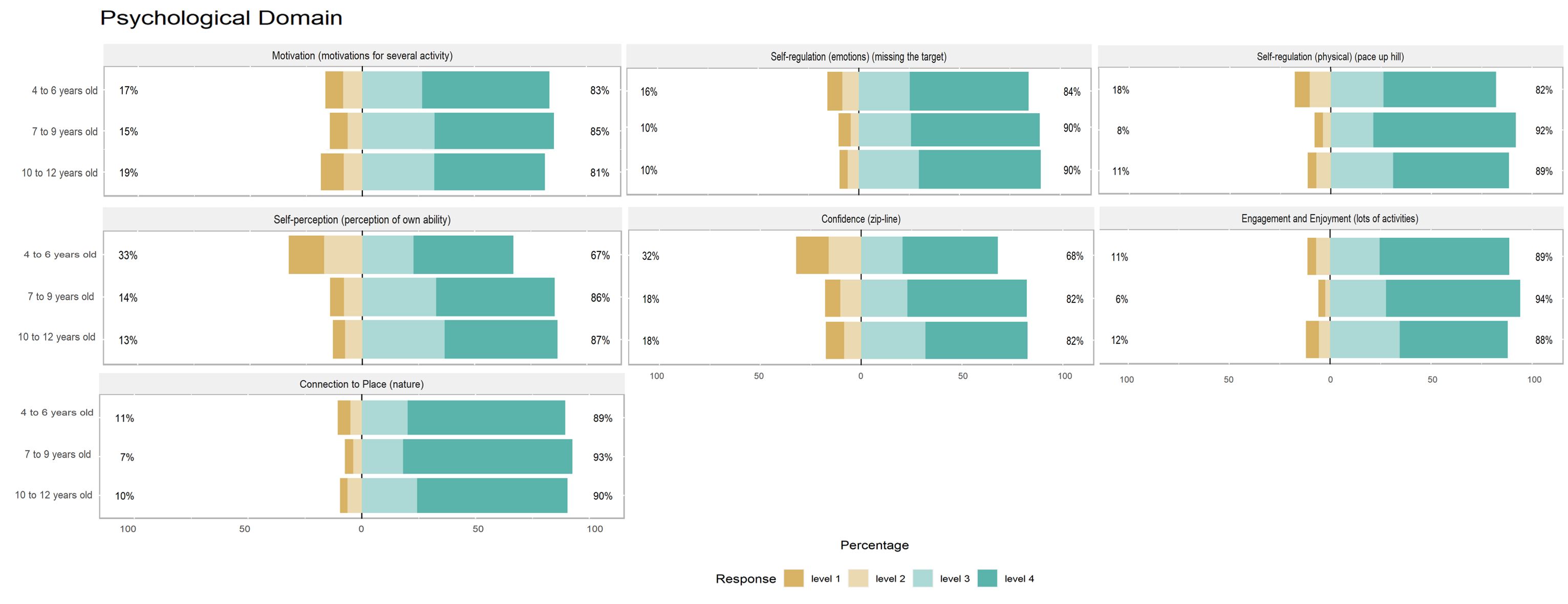
**

**Supplementary Figure 3**

*Chart of* *responses (level 1-4, %) for each physical literacy items in the social domain by age groups*


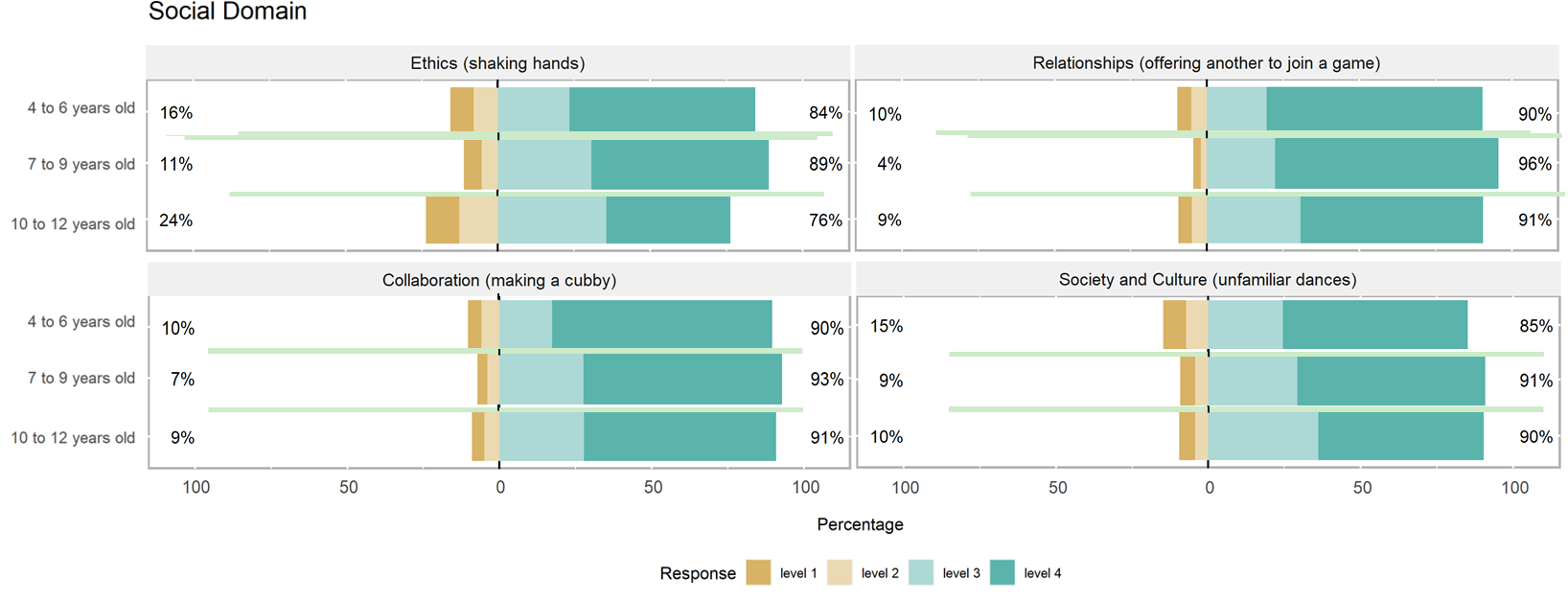


**Supplementary Figure 4**

*Chart of responses (level 1-4, %) for each physical literacy items in the cognitive domain by age groups*

**
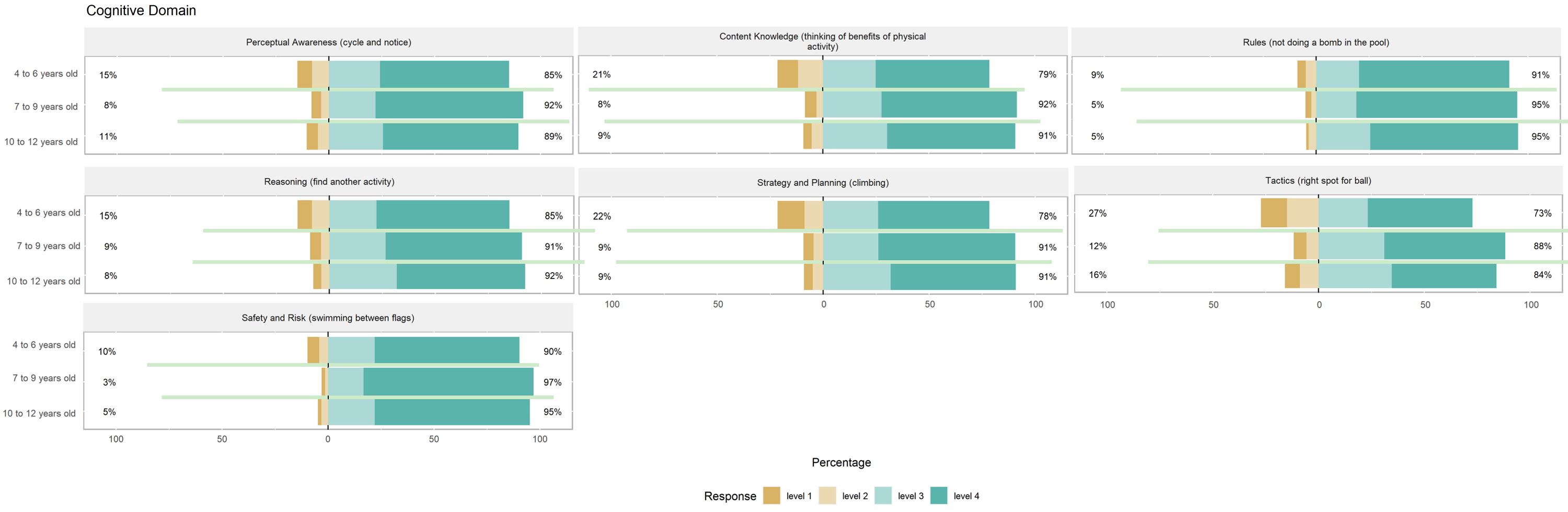
**
