# Supplementary material for: The validity of the Physical Literacy in Children Questionnaire in children aged 4 to 12
Source: BMC Public Health. 2024 Mar 21;24:869. doi: 10.1186/s12889-024-18343-x (PMC10956319; doi:10.1186/s12889-024-18343-x)
Supplement: Supplementary file 1 — Supplementary Material 1. [file 12889_2024_18343_MOESM1_ESM.zip › Supp_table 1.docx]

**Supplementary Table 1**

Distribution of responses for each physical literacy item (level 1-4, %) and by sex (girls, n = 871; boys, n = 999)

| Item (item description) | Total | | | |  | Girls | | | |  | Boys | | | |  | Girls VS Boys | |
| --- | --- | --- | --- | --- | --- | --- | --- | --- | --- | --- | --- | --- | --- | --- | --- | --- | --- |
|  | L1 | L2 | L3 | L4 |  | L1 | L2 | L3 | L4 |  | L1 | L2 | L3 | L4 |  | *χ^2^* | *P* |
| ***Physical domain*** |  |  |  |  |  |  |  |  |  |  |  |  |  |  |  |  |  |
| *Movement Skills* (hopping) | 9.3 | 7.6 | 27.0 | 56.0 |  | 9.0 | 6.8 | 28.7 | 55.6 |  | 9.6 | 8.4 | 25.5 | 56.5 |  | 3.645 | 0.302 |
| *Moving with Equipment* (skateboarding) | 29.6 | 22.2 | 22.8 | 25.3 |  | 32.0 | 23.4 | 22.5 | 22.0 |  | 27.5 | 21.2 | 23.0 | 28.2 |  | 11.276 | 0.010 |
| *Object Manipulation* (overarm throwing) | 14.3 | 11.8 | 27.3 | 46.6 |  | 19.1 | 13.1 | 28.8 | 39.0 |  | 10.1 | 10.7 | 25.9 | 53.3 |  | 49.919 | 0.000 |
| *Cardiovascular Endurance* (long-distance run) | 13.6 | 12.5 | 27.0 | 46.9 |  | 15.3 | 13.5 | 26.5 | 44.7 |  | 12.2 | 11.6 | 27.3 | 48.8 |  | 6.436 | 0.092 |
| *Muscular Endurance* (hanging) | 19.2 | 19.0 | 26.6 | 35.2 |  | 21.4 | 19.5 | 26.4 | 32.7 |  | 17.3 | 18.5 | 26.7 | 37.4 |  | 7.151 | 0.067 |
| *Coordination* (skipping) | 12.9 | 11.4 | 25.7 | 49.9 |  | 11.7 | 11.4 | 26.1 | 50.9 |  | 14.0 | 11.4 | 25.4 | 49.1 |  | 2.255 | 0.521 |
| *Stability/Balance* (balancing on a rock) | 9.7 | 9.4 | 27.9 | 53.0 |  | 10.8 | 9.3 | 28.2 | 51.7 |  | 8.7 | 9.5 | 27.6 | 54.2 |  | 2.716 | 0.438 |
| *Flexibility* (touching toes) | 10.2 | 10.4 | 23.4 | 56.0 |  | 7.1 | 7.2 | 21.6 | 64.1 |  | 12.9 | 13.1 | 25.0 | 48.9 |  | 52.144 | 0.000 |
| *Agility* (dodging in a game) | 7.1 | 7.4 | 26.2 | 59.4 |  | 8.2 | 7.6 | 29.2 | 55.1 |  | 6.1 | 7.2 | 23.6 | 63.1 |  | 13.251 | 0.004 |
| *Strength* (lifting a rock) | 15.0 | 12.9 | 28.9 | 43.2 |  | 20.8 | 17.1 | 28.4 | 33.8 |  | 9.9 | 9.3 | 29.4 | 51.4 |  | 92.158 | 0.000 |
| *Reaction Time* (running at the starter gun) | 7.0 | 8.1 | 27.5 | 57.4 |  | 7.1 | 8.4 | 30.5 | 54.0 |  | 6.8 | 7.8 | 24.9 | 60.5 |  | 9.003 | 0.029 |
| *Speed* (running in a race) | 10.7 | 11.4 | 28.2 | 49.7 |  | 11.8 | 12.9 | 30.5 | 44.8 |  | 9.8 | 10.1 | 26.1 | 54.0 |  | 15.951 | 0.001 |
| ***Psychological domain*** |  |  |  |  |  |  |  |  |  |  |  |  |  |  |  |  |  |
| *Motivation* (motivations for several activity) | 8.6 | 7.8 | 30.2 | 53.4 |  | 10.3 | 8.6 | 31.6 | 49.5 |  | 7.1 | 7.1 | 28.9 | 56.9 |  | 12.786 | 0.005 |
| *Self-regulation (emotions)* (missing the target) | 5.9 | 6.0 | 26.8 | 61.2 |  | 6.3 | 6.3 | 29.6 | 57.7 |  | 5.6 | 5.8 | 24.3 | 64.3 |  | 8.691 | 0.034 |
| *Self-regulation (physical)* (pace up hill) | 5.3 | 7.3 | 25.8 | 61.6 |  | 5.2 | 8.8 | 28.8 | 57.2 |  | 5.5 | 5.9 | 23.1 | 65.5 |  | 16.654 | 0.001 |
| *Self-perception* (perception of own ability) | 9.4 | 11.3 | 30.3 | 49.0 |  | 9.5 | 10.7 | 31.8 | 48.0 |  | 9.3 | 11.8 | 29.0 | 49.8 |  | 2.063 | 0.559 |
| *Confidence* (zip-lining) | 11.1 | 11.9 | 24.7 | 52.4 |  | 12.1 | 13.0 | 24.7 | 50.3 |  | 10.2 | 11.0 | 24.6 | 54.2 |  | 4.263 | 0.234 |
| *Engagement and Enjoyment* (lots of activities) | 4.6 | 5.0 | 28.5 | 61.9 |  | 5.1 | 4.8 | 31.1 | 59.0 |  | 4.2 | 5.1 | 26.2 | 64.5 |  | 6.935 | 0.074 |
| *Connection to Place* (nature) | 4.3 | 5.0 | 20.6 | 70.2 |  | 3.7 | 5.1 | 21.9 | 69.3 |  | 4.8 | 4.9 | 19.4 | 70.9 |  | 2.989 | 0.393 |
| ***Social domain*** |  |  |  |  |  |  |  |  |  |  |  |  |  |  |  |  |  |
| *Ethics* (shaking hands) | 8.0 | 8.4 | 29.5 | 54.1 |  | 8.3 | 8.4 | 31.5 | 51.9 |  | 7.7 | 8.4 | 27.8 | 56.1 |  | 3.749 | 0.290 |
| *Relationships* (offering another to join a game) | 3.7 | 4.0 | 23.7 | 68.5 |  | 4.5 | 3.8 | 25.1 | 66.6 |  | 3.1 | 4.2 | 22.5 | 70.2 |  | 4.766 | 0.190 |
| *Collaboration* (making a cubby) | 3.9 | 5.0 | 23.9 | 67.2 |  | 4.2 | 4.8 | 25.1 | 65.8 |  | 3.6 | 5.1 | 22.8 | 68.5 |  | 2.116 | 0.549 |
| *Society and Culture* (unfamiliar dances) | 6.0 | 5.3 | 29.5 | 59.2 |  | 5.1 | 4.8 | 30.1 | 60.0 |  | 6.8 | 5.8 | 28.9 | 58.5 |  | 3.643 | 0.303 |
| ***Cognitive domain*** |  |  |  |  |  |  |  |  |  |  |  |  |  |  |  |  |  |
| *Perceptual Awareness* (cycle and notice) | 5.7 | 5.7 | 23.8 | 64.9 |  | 5.5 | 6.8 | 25.0 | 62.7 |  | 5.8 | 4.7 | 22.7 | 66.8 |  | 5.820 | 0.121 |
| *Content Knowledge* (thinking of benefits  of physical activity) | 6.5 | 6.8 | 27.4 | 59.2 |  | 6.5 | 8.2 | 30.4 | 54.9 |  | 6.5 | 5.7 | 24.8 | 63.0 |  | 14.523 | 0.002 |
| *Rules* (not doing a bomb in the pool) | 2.7 | 3.6 | 21.3 | 72.4 |  | 1.7 | 2.6 | 20.4 | 75.2 |  | 3.6 | 4.4 | 22.1 | 69.9 |  | 12.527 | 0.006 |
| *Reasoning* (find another activity) | 5.3 | 5.5 | 26.5 | 62.7 |  | 4.9 | 5.9 | 28.0 | 61.2 |  | 5.7 | 5.2 | 25.1 | 64.0 |  | 2.908 | 0.406 |
| *Strategy and Planning* (climbing) | 7.4 | 6.3 | 27.6 | 58.7 |  | 7.3 | 6.9 | 30.2 | 55.6 |  | 7.5 | 5.7 | 25.4 | 61.4 |  | 7.548 | 0.056 |
| *Tactics* (right spot for ball) | 8.6 | 9.9 | 29.3 | 52.2 |  | 9.8 | 11.7 | 32.1 | 46.4 |  | 7.6 | 8.4 | 26.7 | 57.3 |  | 22.817 | 0.000 |
| *Safety and Risk* (swimming between flags) | 3.1 | 2.9 | 20.2 | 73.9 |  | 2.4 | 2.8 | 21.7 | 73.1 |  | 3.7 | 3.0 | 18.8 | 74.5 |  | 4.634 | 0.201 |

*Note.* Level 1 (L1), Level 2 (L1), Level 3 (L3), Level 4 (L4). χ^2^ statistic was calculated to identify sex-based differences in the responses to each item.
