# Supplementary material for: The validity of the Physical Literacy in Children Questionnaire in children aged 4 to 12
Source: BMC Public Health. 2024 Mar 21;24:869. doi: 10.1186/s12889-024-18343-x (PMC10956319; doi:10.1186/s12889-024-18343-x)
Supplement: Supplementary file 1 — Supplementary Material 1. [file 12889_2024_18343_MOESM1_ESM.zip › Supp_table 2.docx]

**Supplementary Table 2**

Distribution of responses (level 1-4, %) for each physical literacy items by age groups

| Items (item description) | A  *n* = 676 | | | | B  *n* = 654 | | | | C  *n* = 540 | | | | Age group difference | | Post-hoc |
| --- | --- | --- | --- | --- | --- | --- | --- | --- | --- | --- | --- | --- | --- | --- | --- |
|  | L1 | L2 | L3 | L4 | L1 | L2 | L3 | L4 | L1 | L2 | L3 | L4 | χ^2^ | *P* |  |
| **Physical domain** |  |  |  |  |  |  |  |  |  |  |  |  |  |  |  |
| *Movement Skills* (hopping) | 12.3 | 9.6 | 25.0 | 53.1 | 7.3 | 5.4 | 24.5 | 62.8 | 8.0 | 8.0 | 32.6 | 51.5 | 20.563 | 0.000 | B>A C>A |
| *Moving with Equipment* (skateboarding) | 33.9 | 14.5 | 21.7 | 29.9 | 27.1 | 29.5 | 21.1 | 22.3 | 27.4 | 23.1 | 26.1 | 23.3 | 9.484 | 0.009 | B<A |
| *Object Manipulation* (overarm throwing) | 15.4 | 13.2 | 22.9 | 48.5 | 9.9 | 8.7 | 28.7 | 52.6 | 18.1 | 13.9 | 30.9 | 37.0 | 30.773 | 0.000 | B>A B>C |
| *Cardiovascular Endurance* (long-distance run) | 9.5 | 10.2 | 24.0 | 56.4 | 12.1 | 9.5 | 26.8 | 51.7 | 20.7 | 19.1 | 30.9 | 29.3 | 74.027 | 0.000 | A>C B>C |
| *Muscular Endurance* (hanging) | 22.3 | 19.7 | 22.5 | 35.5 | 16.2 | 17.6 | 26.1 | 40.1 | 18.9 | 19.8 | 32.2 | 29.1 | 9.603 | 0.008 | B>A |
| *Coordination* (skipping) | 23.7 | 17.3 | 20.1 | 38.9 | 6.0 | 6.0 | 24.9 | 63.1 | 8.0 | 10.6 | 33.7 | 47.8 | 166.295 | 0.000 | B>A B>C C>A |
| *Stability/Balance* (balancing on a rock) | 13.3 | 14.3 | 23.2 | 49.1 | 8.1 | 6.9 | 27.7 | 57.3 | 7.0 | 6.3 | 34.1 | 52.6 | 50.884 | 0.000 | B>A C>A |
| *Flexibility* (touching toes) | 8.6 | 7.1 | 20.9 | 63.5 | 9.9 | 10.7 | 22.2 | 57.2 | 12.6 | 14.1 | 28.1 | 45.2 | 22.163 | 0.000 | A>C B>C |
| *Agility* (dodging in a game) | 11.4 | 12.1 | 23.5 | 53.0 | 4.9 | 4.0 | 25.4 | 65.7 | 4.3 | 5.6 | 30.6 | 59.6 | 70.906 | 0.000 | B>A C>A |
| *Strength* (lifting a rock) | 17.6 | 16.1 | 24.3 | 42.0 | 12.5 | 10.4 | 27.1 | 50.0 | 14.6 | 12.0 | 37.0 | 36.3 | 19.827 | 0.000 | B>A C>A |
| *Reaction Time* (running at the starter gun) | 6.4 | 9.0 | 24.1 | 60.5 | 6.1 | 3.4 | 27.1 | 63.5 | 8.7 | 12.6 | 32.4 | 46.3 | 32.449 | 0.000 | B>A B>C A>C |
| *Speed* (running in a race) | 7.4 | 9.5 | 27.2 | 55.9 | 9.6 | 8.3 | 27.1 | 55.0 | 16.3 | 17.6 | 30.7 | 35.4 | 61.012 | 0.000 | A>C B>C |
| **Psychological domain** |  |  |  |  |  |  |  |  |  |  |  |  |  |  |  |
| *Motivation* (motivations for several activity) | 8.0 | 8.6 | 26.6 | 56.8 | 8.0 | 6.6 | 32.3 | 53.2 | 10.2 | 8.3 | 32.0 | 49.4 | 3.454 | 0.178 |  |
| *Self-regulation (emotions)* (missing the target) | 7.4 | 8.3 | 25.3 | 59.0 | 6.0 | 4.1 | 25.8 | 64.1 | 4.1 | 5.6 | 29.8 | 60.6 | 13.820 | 0.001 | B>A C>A |
| *Self-regulation (physical)* (pace up hill) | 7.4 | 10.5 | 26.2 | 55.9 | 4.1 | 4.0 | 21.1 | 70.8 | 4.3 | 7.2 | 30.9 | 57.6 | 29.816 | 0.000 | B>A C>A |
| *Self-perception* (perception of own ability) | 15.8 | 17.0 | 22.8 | 44.4 | 6.1 | 8.3 | 32.9 | 52.8 | 5.4 | 7.8 | 36.7 | 50.2 | 95.421 | 0.000 | B>A C>A |
| *Confidence* (zip-lining) | 16.1 | 16.1 | 20.6 | 47.2 | 7.5 | 10.4 | 22.9 | 59.2 | 9.1 | 8.5 | 31.9 | 50.6 | 51.216 | 0.000 | B>A C>A |
| *Engagement and Enjoyment* (lots of activities) | 4.3 | 7.0 | 24.6 | 64.2 | 3.4 | 2.4 | 27.7 | 66.5 | 6.5 | 5.6 | 34.4 | 53.5 | 16.661 | 0.000 | B>A B>C |
| *Connection to Place* (nature) | 5.6 | 5.0 | 20.1 | 69.2 | 3.7 | 3.8 | 18.0 | 74.5 | 3.3 | 6.3 | 24.3 | 66.1 | 4.079 | 0.130 |  |
| **Social domain** |  |  |  |  |  |  |  |  |  |  |  |  |  |  |  |
| *Ethics* (shaking hands) | 7.5 | 8.0 | 23.5 | 60.9 | 5.8 | 5.4 | 30.7 | 58.1 | 11.1 | 12.6 | 35.6 | 40.7 | 34.528 | 0.000 | A>C B>C |
| *Relationships* (offering another to join a game) | 4.4 | 5.2 | 19.5 | 70.9 | 2.4 | 2.0 | 22.3 | 73.2 | 4.4 | 5.0 | 30.7 | 59.8 | 15.508 | 0.000 | B>A B>C |
| *Collaboration* (making a cubby) | 4.4 | 5.9 | 17.3 | 72.3 | 3.2 | 4.0 | 27.5 | 65.3 | 4.1 | 5.0 | 27.8 | 63.1 | 4.162 | 0.125 |  |
| *Society and Culture* (unfamiliar dances) | 7.5 | 7.2 | 24.4 | 60.8 | 4.9 | 4.3 | 29.2 | 61.6 | 5.4 | 4.3 | 36.1 | 54.3 | 12.641 | 0.002 | B>A C>A |
| **Cognitive domain** |  |  |  |  |  |  |  |  |  |  |  |  |  |  |  |
| *Perceptual Awareness* (cycle and notice) | 7.0 | 8.0 | 24.1 | 60.9 | 4.6 | 3.7 | 22.0 | 69.7 | 5.4 | 5.2 | 25.6 | 63.9 | 15.235 | 0.000 | B>A |
| *Content Knowledge*  (thinking of benefits of physical activity) | 9.8 | 11.7 | 24.9 | 53.7 | 5.4 | 3.1 | 27.7 | 63.9 | 3.9 | 5.4 | 30.4 | 60.4 | 59.876 | 0.000 | B>A C>A |
| *Rules* (not doing a bomb in the pool) | 4.0 | 4.9 | 20.3 | 70.9 | 2.8 | 2.3 | 19.0 | 76.0 | 1.1 | 3.5 | 25.6 | 69.8 | 11.874 | 0.003 | B>A C>A |
| *Reasoning* (find another activity) | 6.8 | 8.3 | 22.2 | 62.7 | 5.2 | 4.0 | 26.5 | 64.4 | 3.7 | 3.9 | 31.9 | 60.6 | 20.369 | 0.000 | B>A C>A |
| *Strategy and Planning* (climbing) | 12.7 | 8.9 | 25.9 | 52.5 | 4.7 | 4.6 | 26.0 | 64.7 | 4.1 | 5.0 | 31.9 | 59.1 | 56.048 | 0.000 | B>A C>A |
| *Tactics* (right spot for ball) | 12.3 | 14.9 | 23.2 | 49.6 | 6.1 | 5.7 | 31.0 | 57.2 | 7.0 | 8.9 | 34.6 | 49.4 | 55.946 | 0.000 | B>A C>A |
| *Safety and Risk* (swimming between flags) | 5.6 | 4.1 | 22.0 | 68.2 | 1.7 | 1.4 | 16.7 | 80.3 | 1.7 | 3.1 | 22.0 | 73.1 | 28.403 | 0.000 | B>A C>A |

*Note*. Level 1 (L1), Level 2 (L1), Level 3 (L3), Level 4 (L4). A = 4 to 6 years. B = 7 to 9 years. C = 10 to 12 years. The post-hoc comparison results for each item with higher levels of child perception (the sum of the proportions of levels 3 and levels 4) were presented in this table.
