# Supplementary material for: The validity of the Physical Literacy in Children Questionnaire in children aged 4 to 12
Source: BMC Public Health. 2024 Mar 21;24:869. doi: 10.1186/s12889-024-18343-x (PMC10956319; doi:10.1186/s12889-024-18343-x)
Supplement: Supplementary file 1 — Supplementary Material 1. [file 12889_2024_18343_MOESM1_ESM.zip › Supp_table 3.docx]

**Supplementary Table 3**

Goodness of fit statistics for the hypothesized CFA SEM PL model also divided by sex and age group

| Model | χ^2^ | *df* | *P* | CFI | TLI | RMSEA [90%CI] |
| --- | --- | --- | --- | --- | --- | --- |
| Model | 1685.673 | 399 | <0.001 | 0.954 | 0.950 | 0.042 [0.039, 0.044] |
| ***Sex*** |  |  |  |  |  |  |
| Model _Boys_ | 916.229 | 399 | <0.001 | 0.968 | 0.965 | 0.036 [0.033, 0.039] |
| Model _Girls_ | 1092.970 | 399 | <0.001 | 0.941 | 0.936 | 0.045 [0.042, 0.048] |
| ***Age group*** |  |  |  |  |  |  |
| Model _A_ | 973.519 | 399 | <0.001 | 0.929 | 0.923 | 0.046 [0.042,0.050] |
| Model _B_ | 723.516 | 399 | <0.001 | 0.968 | 0.965 | 0.035 [0.031,0.039] |
| Model _C_ | 869.762 | 399 | <0.001 | 0.956 | 0.952 | 0.047 [0.043,0.051] |

*Note*. A = 4 to 6 years. B = 7 to 9 years. C = 10 to 12 years. χ^2^= chi-square. *df* = degrees of freedom. CFI = comparative fit index. TLI = Tucker-Lewis index. RMSEA = Root Mean Square Error of Approximation. Probability level *p* < 0.05
